# Editorial: Understanding Molecular Mechanisms in Diabetic Cardiomyopathy (DCM)

**DOI:** 10.3389/fcvm.2022.965650

**Published:** 2022-06-30

**Authors:** Sakthijothi Muthu, Vijayakumar Sukumaran, Venkatesh Sundararajan

**Affiliations:** ^1^Department of Physiology and Pharmacology, School of Medicine, West Virginia University, Morgantown, WV, United States; ^2^Biomedical Research Center, Qatar University, Doha, Qatar

**Keywords:** cardiovascular disease, diabetes, cardiomyopathy, mitochondria, oxidative stress, hypertrophy

Cardiovascular disease is the leading cause of death worldwide, and diabetes, the most common metabolic syndrome, causes a 2–5 times higher risk for heart disease (1). Specifically, cardiovascular complications in people with diabetes mellitus (DM) are 2-3-fold more elevated than in non-diabetic counterparts, leading to a higher chance of causing diabetic cardiomyopathy (DCM). DCM is a prominent disease in people with DM, and molecular mechanisms that drive DCM are not fully understood as the disease itself is multifactorial and challenging potential treatment options (2). Therefore, this Research Topic is focused on understanding the molecular mechanism(s) contributing to DCM. The Research Topic comprised review and research articles from different experts working on DCM. The Research Topic of original research and review topics highlights the multifactorial molecular mechanisms involved in the DCM and some recent therapeutic advances in preclinical approaches at various levels in the pipeline.

One of the major issues during cardiac pathology is impaired protein quality of the myocardium, which is more prone to damage under diabetic conditions (3). The review article by Kaur et al. comprehensively focused on the protein quality control (PQC) system that appears to play a vital role in maintaining cardiomyocyte viability under physiological and pathological conditions. Protein homeostasis is preserved by the molecular process of protein translation, degradation of misfolded or unfolded proteins, recycling of amino acids, and disposing of toxic substances by various quality control pathways. These pathways include unfolded protein response (UPR), ubiquitin-protease system (UPS), autophagy and mitophagy. In addition, the role of unfolded protein response (UPR) in Endoplasmic reticulum (UPR^ER^) and mitochondria (UPR^Mt^), and the molecules involved in these pathways and their contribution to DCM are discussed. Compromised PQC leads to impaired cellular homeostasis that results in the aggregation of misfolded proteins and toxic substances, which provokes the onset of heart failure in diabetes. Therefore, the finely tuned manipulation of PQC in the myocardium is essential to maintain cellular equilibrium in response to the diabetic condition and may be a promising therapeutic strategy for the betterment of cardiac complications in diabetes patients.

Sudden cardiac death (SCD) in patients with heart failure preserved with ejection fraction (HFpEF) is another major problem in people with DM, as these patients are more prone to morbidity and cardiac mortality (4). Patel et al. found that the upregulation of Osteopontin (OPN) shows adverse cardiac remodeling, whereas OPN knockdown reduced HFpEF, improved glucose tolerance, and reduced insulin resistance in mice. Therefore, the expression level of OPN, along with its correlated proteins such as low-density lipoprotein receptor (LDLR), dynamin2 (DNM2), fibronectin-1(FN1), and 2-oxoglutarate dehydrogenase-like (OGDHL) are predicted to serve as a potential risk marker for SCD in DCM. Authors also find that dysregulated expression of these proteins in patients with DM and HFpEF who experienced SCD may serve as a predictive plasma biomarker that determines whether its expression level alters SCD risk in patients with DM and HFpEF. As they are secreted into plasma, OPN and LDLR levels could serve as potential biomarkers in DCM that require further investigation.

A common feature of the diabetic heart is the excess production of ROS due to metabolic inflexibility that accelerates the progression of DCM, which ultimately triggers mitochondrial dysfunction. Parker et al. established a model to study DCM, based on the mitochondrial structure, replicating diabetic myocardium that could be useful to research mitochondrial targets in DCM. In this work, DCM was induced by low-dose streptozotocin (STZ) and a high-fat diet that resulted in a spectrum of mitochondrial changes observed in diabetes. They found that this model replicated several features of DCM, such as decreased mitochondrial area, reduced complex-II dependent oxygen consumption, and increased levels of Complex-III and V proteins.

Cardiovascular disease is the major obstacle to diabetes-associated complications, besides creating mechanical abnormalities of the myocardium. Another prominent feature of diabetic myocardium is the altered electrical remodeling causing cardiac arrhythmia. In this regard, Gallego et al. presented a comprehensive review on the importance of considering the electrical features of the myocardium when treated for diabetes. They provided details of the underlying cellular level mechanisms that alter cardiac ion channels, regulatory proteins, and a subsequent change in sodium, calcium, and potassium currents. Altogether, this collectively lengthens the QT interval duration, increasing the risk of developing life-threatening ventricular arrhythmias and sudden death. As QT duration strongly correlates with the risk of developing ventricular tachycardia followed by ventricular fibrillation, the thorough QT/QTc analysis evaluates QT interval prolongation that may act as a qualitative marker for proarrhythmic risk. Diabetic patients treated with the glucose-lowering drug showed prolonged QTc intervals, indicating that strict glycemic control is insufficient to normalize the electrophysiological disturbances. Careful studies are required to elucidate if cardio protection includes electrical remodeling and prolonged repolarization, as authors predict that the mechanism of cardioprotection might involve a reduction of arrhythmia.

A review article by Kassab et al. focused on the role of mitochondrial outer membrane protein, Miro1, which mediates the movement of mitochondria along the microtubules. Authors emphasized the importance of mitochondrial dynamic, motility, shape, and structure in the diabetic myocardium as reports show smaller mitochondria associated with diabetic myocardium. They also pointed out in this review that HF and diabetes are recognized with enhanced activation of the NLRP3 inflammasome that links microtubule organization, inflammation, and the association to mitochondrial motility, which may lead to novel therapeutic approaches toward the mitochondrial-microtubule-inflammatory axis. Furthermore, the expression of Miro1 and whether it influences the processes of fission and fusion for mitophagy in the heart remains unclear, which led to this review. Based on the existing literature, the authors suggest a possible unifying mechanism linking impaired mitophagy, the MT network, and the inflammatory response to arrested mitochondrial movement. As the causative agents and mechanisms of mitochondrial dysfunction and impaired motility are discovered, new promising treatment therapies may emerge to promote better cardiac outcomes in DCM/HF.

In another interesting study, Huang et al. targeted a specific protein, P2X7R, whose role in diabetic myocardium is not studied but predicted to play an essential role. When they treated mice with a P2X7R inhibitor (A438079), they reduced myocardial hypertrophy, fibrosis, and apoptosis and improved cardiac function. Mechanistically they showed that P2X7R plays an essential role in the pathogenesis of STZ-induced diabetic cardiac damage and remodeling through the PKCβ/ERK axis and suggest that P2X7R may be a potential target in the treatment of DCM. Although one of the critical features of DCM is cardiac fibrosis (CF), which is still not successfully targeted in treating heart failure, Lin et al. showed that inhibition of sodium-dependent glucose transporter 1 (SGLT1) attenuates cardiomyocyte apoptosis and delays the development of DCM. Further, they evaluated the changes in the expression of SGLT1 in the progression of diabetic cardiac fibrosis. They identified a significant increase in SGLT1 expression in the diabetic heart, concluding that SGLT1 is involved in cardiac fibrosis *via* the p38 and ERK1/2 signaling pathways.

Shraim et al. reviewed the Epidermal Growth Factor Receptor (EGFR/ErbB/HER) family of tyrosine kinases in diabetes myocardium as chronic dysregulation of EGFR may act as mediating diabetes-induced cardiovascular pathology. Authors examined their potential interplay with the Renin-Angiotensin-Aldosterone System heptapeptide, Angiotensin-(1-7), as well the arachidonic acid metabolite, 20-HETE (20-hydroxy-5, 8, 11, 14-eicosatetraenoic acid). Its greater understanding of other critical modulators of cardiovascular function could facilitate the development of novel therapeutic strategies for treating diabetes-induced cardiovascular complications.

Muñoz-Córdova et al. critically discussed the mechanism of diabetic mediated cardiac dysfunction by highlighting three critical areas: inflammatory signaling, mitochondrial alterations, and autophagic flux, their contribution to the pathogenesis, and their potential as pharmacological targets. Here, they critically discussed the mechanisms by highlighting the latest evidence, their contribution to the pathogenesis, and their potential as pharmacological targets. Though plenty of antidiabetic agents benefit HF, there is scarce information available for DCM. Authors cited the combined effect of drugs such as liraglutide and dapagliflozin that showed improved diastolic function and regression of left ventricular mass in patients with T2DM. On the other hand, Sitagliptin prevents the exacerbation of DCM in T2DM when it is used together with other antidiabetic drugs. Therefore, the authors highlight the importance of considering already approved therapies in searching for new DCM as a study of the mechanism can lead to discovering new molecules with better therapeutical properties.

Association of epigenetics and various factors and how those contributes to the development of DCM is comprehensively reviewed by Mittal et al. They discussed different epigenetic mechanisms such as histone modifications (acetylation and methylation), DNA methylation and non-coding RNA in modulating molecular pathways regulating the expression of important molecules. Authors cleverly explored the disease with complex etiology and cumulative effects of crosstalk between genetic and epigenetic factors, which has several inducers such as ROS-mediated oxidative stress, hyperglycemic conditions, cytokines-mediated inflammation, cell death, and epigenetic regulation of dysregulated molecular pathways induced by these mediators. They also discussed the possibility of inhibiting HDACs as promising therapeutic targets for DCM. In another review article, Mohan et al. discussed and evaluated critical clinical studies that studied the effect of drugs such as allopurinol, SGLT2 inhibitor, and metformin in reverting left ventricular hypertrophy patients with and without T2DM.

Interestingly, Liu et al. demonstrated that a combinational exposure of bisphenol A (BPA) and a high-fat diet (HFD) in female mice during the perinatal period can cause susceptibility to insulin resistance, obesity, impaired glucose tolerance, and increased blood pressure, cardiac hypertrophy, and impaired endothelial function in their F2 offspring. In addition, these inherited transgenerational abnormalities showed a sex-specific pattern. They strongly recommend adjusting lifestyle and alleviating exposure to environmental EDSs during pregnancy to reduce the risk of metabolic and cardiovascular diseases such as DCM in the offspring.

In conclusion, this Research Topic emphasizes various molecular mechanisms involved in DCM development and explores potential therapeutic targets against DCM. Although DCM is a multifactorial complex metabolic disease, this Research Topic will be an essential key to conceptual advancement in the field of DCM.

## Author Contributions

All authors listed have made a substantial, direct, and intellectual contribution to the work and approved it for publication.

## Funding

VS laboratory is partially supported by the American Heart Association grants (20CDA35260096 and 20TPA3542000).

## Conflict of Interest

The authors declare that the research was conducted in the absence of any commercial or financial relationships that could be construed as a potential conflict of interest.

## Publisher's Note

All claims expressed in this article are solely those of the authors and do not necessarily represent those of their affiliated organizations, or those of the publisher, the editors and the reviewers. Any product that may be evaluated in this article, or claim that may be made by its manufacturer, is not guaranteed or endorsed by the publisher.

## References

[1] Martin-Timon I Sevillano-Collantes C Segura-Galindo A Del Canizo-Gomez FJ. Type 2 diabetes and cardiovascular disease: have all risk factors the same strength? World J Diabetes. (2014) 5:444–70. 10.4239/wjd.v5.i4.444 25126392 PMC4127581

[2] Low Wang CC Hess CN Hiatt WR Goldfine AB. Clinical update: cardiovascular disease in diabetes mellitus: atherosclerotic cardiovascular disease and heart failure in type 2 diabetes mellitus - mechanisms, management, and clinical considerations. Circulation. (2016) 133:2459–502. 10.1161/CIRCULATIONAHA.116.022194 27297342 PMC4910510

[3] Baseler WA Dabkowski ER Williamson CL Croston TL Thapa D Powell MJ. Proteomic alterations of distinct mitochondrial subpopulations in the type 1 diabetic heart: contribution of protein import dysfunction. Am J Physiol Regul Integr Comp Physiol. (2011) 300:R186–200. 10.1152/ajpregu.00423.2010 21048079 PMC3043804

[4] Walker AM Cubbon RM. Sudden cardiac death in patients with diabetes mellitus and chronic heart failure. Diab Vasc Dis Res. (2015) 12:228–33. 10.1177/1479164115573225 25861812

